# Effect of Sucrose Concentration on Sucrose-Dependent Adhesion and Glucosyltransferase Expression of *S. mutans* in Children with Severe Early-Childhood Caries (S-ECC)

**DOI:** 10.3390/nu6093572

**Published:** 2014-09-09

**Authors:** Wei Zhao, Wenqing Li, Jiacheng Lin, Zhuoyu Chen, Dongsheng Yu

**Affiliations:** Guanghua School of Stomatology, Guangdong Provincial Key Laboratory of Stomatology, Sun Yat-sen University, Guangzhou 510055, China; E-Mails: zhaowei3@mail.sysu.edu.cn (W.Z.); wendysums@hotmail.com (W.L.); linjiach@mail.sysu.edu.cn (J.L.); chzhuoy@mail2.sysu.edu.cn (Z.C.)

**Keywords:** sucrose, severe early childhood caries, *S. mutans*, glucosyltransferases, genotype

## Abstract

Sucrose, extracellular polysaccharide, and glucosyltransferases (GTFs) are key factors in sucrose-dependent adhesion and play important roles in the process of severe early-childhood caries (S-ECC). However, whether sucrose concentration regulates gtf expression, extracellular polysaccharide synthesis, and sucrose-dependent adhesion is related to the different genotypes of *S. mutans* isolated from ECC in children and still needs to be investigated. In this study, 52 strains of *S. mutans* were isolated from children with S-ECC and caries-free (CF) children. Water-insoluble glucan (WIG) synthesis was detected by the anthrone method, adhesion capacity by the turbidimetric method, and expression of gtf by RT-PCR in an *in vitro* model containing 1%–20% sucrose. The genotypes of *S. mutans* were analyzed by AP-PCR. The results showed that WIG synthesis, adhesion capacity, and gtf expression increased significantly when the sucrose concentration was from 1% to 10%. WIG synthesis and gtfB as well as gtfC expression of the 1% and 5% groups were significantly lower than those of the 10% and 20% groups (*p* < 0.05). There were no significant differences between the 10% and 20% groups. The fingerprints of *S. mutans* detected from individuals in the S-ECC group exhibited a significant difference in diversity compared with those from CF individuals (*p* < 0.05). Further, the expression of gtfB and gtfC in the S-ECC group was significantly different among the 1- to 5-genotype groups (*p* < 0.05). It can be concluded that sucrose-dependent adhesion might be related to the diversity of genotypes of *S. mutans,* and the 10% sucrose level can be seen as a “turning point” and essential factor for the prevention of S-ECC.

## 1. Introduction

Early-childhood caries (ECC) is defined as “the presence of one or more decayed (non-cavitated or cavitated lesions), missing (due to caries) or filled tooth surfaces” in any primary tooth in a child 71 months old or younger. In children younger than three years old, any sign of smooth-surface caries is indicative of severe early-childhood caries (S-ECC) (American Academy of Pediatric Dentistry, 2003). ECC is a serious public health problem in many developing countries and low socio-economic groups in Western industrialized nations. According to the report of epidemiological studies provided by the Chinese Center for Disease Control and Prevention, the prevalence rate of ECC is as high as 66% in the Chinese mainland. Therefore, assessing, managing, and preventing ECC are important.

ECC is characterized as a carbohydrate-induced infectious bacterial disease that can cause severe damage to primary dentition. Excessive carbohydrate (especially sucrose) uptake and *Streptococcus mutans* (*S. mutans*) are the principal pathogenic factors, according to previous etiologic research [1]. Colonization by *S. mutans* at an early age is considered to correlate with higher caries activity during childhood, and the cariogenicity of these organisms is related, in part, to their ability to colonize and accumulate on tooth surfaces in the presence of sucrose.

Sucrose is the most cariogenic carbohydrate because it is acidogenic and, more importantly, can serve as a substrate for extracellular polysaccharide synthesis by glucosyltransferases (GTFs) of *S. mutans* [2,3]. *In situ* studies have also confirmed that a higher concentration and frequency of sucrose exposure increase extracellular polysaccharide concentration in the biofilm matrix, lower fasting pH values, and enhance enamel demineralization as compared with biofilms formed in the absence of sucrose [4,5,6,7]. Clinical studies have also suggested that synthesis of extracellular polysaccharide is related to caries activity in children [8,9]. Extracellular polysaccharide synthesis by GTFs is essential for the establishment of a matrix that enhances the coherence of bacterial cells and adherence to tooth surfaces [10]. Therefore, it is evident that sucrose, GTFs, and extracellular polysaccharide are key factors involved in the sucrose-dependent adhesion of *S. mutans* and the development of ECC. However, currently, studies of these virulence factors and the relationships of clinical isolates of *S. mutans* from ECC are rare.

A previous study found that differences in caries experience in *S. mutans*-infected children with ECC correlated with the differences in the capacities for extracellular polysaccharide synthesis. The results suggested that *S. mutans* strains appear to differ with regard to their GTFs-mediated virulence. There are several factors that can influence gtf gene transcription, such as carbohydrate availability and source [11,12,13,14,15]. Whether sucrose concentration can regulate gtf expression, extracellular polysaccharide synthesis, and sucrose-dependent adhesion and the nature of the relationship of gtf expression and genotypes of *S. mutans* isolated from children with ECC still need to be investigated.

Thus, the objective of this study was to use an *in vitro* culture model to investigate the effects of diverse levels of sucrose on the synthesis of water-insoluble glucan (WIG), capacity for adhesion, and gtf expression of *S. mutans* isolated from the dental plaque of children with S-ECC and caries-free (CF) children. Furthermore, the genotypes of *S. mutans* and their relationship with gtf expression were also examined.

## 2. Materials and Methods

### 2.1. Study Population

Sixty-seven children, aged 2.7 to 5 years old, from the kindergarten affiliated with Sun Yat-sen University (39 boys and 28 girls) were recruited for this study. They were divided into two groups, 32 CF children and 35 children with S-ECC whose decayed, missing, and filled tooth surface (dmfs) scores were 9.3 ± 5.3. Written informed consent was obtained from all parents and/or caregivers, and the experimental procedures were approved by the Institutional Ethical Committee of the School of Stomatology, Sun Yat-sen University.

### 2.2. Sampling

Pooled samples of dental plaque were taken with sterile dental probes from buccal surfaces of anterior teeth and the first mandibular molar. The samples were immediately placed in sterilized tubes containing PBS with 2% sodium thioglycollate, stored on ice, and transferred to the laboratory within 2 h [16,17].

### 2.3. S. mutans Isolation and Identification

For the detection of *S. mutans*, undiluted samples and 10^−1^–10^−3^ dilutions were cultured on Trypticase Yeast-Extract Cysteine Sucrose Bacitracin (TYCSB) (Difco, Lawrence, Kansas, MI, USA) plates supplemented with 20% sucrose and 0.2U bacitracin mL/L. The plates were incubated at 37 °C for 48 h in an atmosphere of 10% CO_2_, 80% N_2_, and 10% H_2_. The UA159 strain was used as the positive control. In addition, 118 strains of pure *S. mutans* were obtained after morphological, biochemical, and physiological identification. All isolates were subjected to PCR for the identification of *S. mutans*.

### 2.4. Assessment of the Cariogenicity of S. mutans

#### 2.4.1. Culture Conditions

Purified *S. mutans* isolates were cultured in tryptone-soy base medium with 1, 5, 10, and 20% sucrose incubated at 37 °C for 18 h in the previously described anaerobic atmosphere. (The different sucrose concentrations were chosen according to previous studies conducted by J.A. Cury [4,5,6,7].

#### 2.4.2. Adherence Analysis

The sucrose-dependent adherence of *S. mutans* was determined turbidimetrically as follows [18,19]. Purified *S. mutans* was cultured at an angle of 30°. After incubation, culture tubes were vigorously mixed in a vortex mixer for 5 s, and non-adhering cells were transferred to fresh tubes. Aliquots of 3 mL of potassium phosphate buffer (0.05 M, pH 7.0) were added to the first tube and agitated for 5 s, and then the released cells were transferred to a third tube. The second and third tubes were centrifuged for 5 min at 5000 *g*, and the pellets were re-suspended in the same buffer. All test tubes of suspended cells were measured by a spectrophotometer at 550 nm. The percentage of adhered cells was calculated by dividing the cell density of adherent cells by the values of total cell density.

#### 2.4.3. Water-Insoluble Glucan (WIG) Synthesis

The quantities of WIG were measured by the anthrone method [20,21]. The cultured tubes were centrifuged for 5 min at 5000 *g*, and the pellets were re-suspended three times in 5 mL 0.4 mol/L NaOH. Then, the supernatant was collected and merged. A solution of 1 mL of supernatant was added to 3 mL of anthrone reagent. The mixtures were then heated at 95 °C for 6 min, followed by spectrophotometric measurement at 625 nm. The amount of WIG was calculated according to the standard curve.

### 2.5. RNA Extraction and Reverse Transcription

Total RNA extraction was performed as previously described [22]. The integrity of RNA was assessed by agarose gel electrophoresis, and the purity of RNA (OD260/OD280) was measured.

### 2.6. Reverse Transcription and Polymerase Chain-Reaction (RT-PCR)

The reverse transcription of mRNA was performed according to the manual provided for the reverse transcription kit (Promega, San Luis Obispo, CA, USA). The primers were designed to amplify gtfB and gtfC [gtfB, (forward) 5′-AGAATACTGATTGGCTGCG-3′ and (reverse) 5′-AAGCACCTTTACCATAGCG-3′; gtfC, (forward) 5′-AGCAGATTCAACTGACGACC-3′ and (reverse) 5′-ATGCGGAAATAGTCTGACGC-3′]. PCR amplification was performed, and PCR products were analyzed by electrophoresis.

### 2.7. Arbitrarily Primed Polymerase Chain-Reaction (AP-PCR) Analysis

DNA of *S. mutans* was extracted according to the manufacturer’s instructions (Promega, San Luis Obispo, CA, USA). AP-PCR fingerprinting for *S. mutans* was performed with the random primer OPA-02 (5′-TGC CGA GCT G-3′), and PCR products were analyzed by electrophoresis.

### 2.8. Statistical Analysis

Data analyses were performed with SPSS 13.0 (SPSS Inc, Chicago, IL, USA). After a normality test was conducted on the variables of WIG, adhesion ratio, and gtf expression in *S. mutans* isolated from children with S-ECC and CF children, a general linear model was established, and multifactor variance analysis was used to evaluate the effect of sucrose concentration on the sucrose-dependent adhesion of *S. mutans.* Data from different groups of *S. mutans* and different concentrations of sucrose groups were compared by the least-squares-difference statistical method. The significance value was set at 0.05.

## 3. Results

### 3.1. Detection of S. mutans in Children with S-ECC and CF Children

*S. mutans* samples were isolated from 32 of the 35 children with S-ECC (92.4%) and 20 of the 32 CF children (62.5%). The results of the chi-square test revealed that the detection frequencies of *S. mutans* in children with S-ECC were significantly higher than those in CF children (*p* < 0.05). In total, 118 strains of *S. mutans* were obtained from both children with S-ECC and CF children (Table 1).

**Table 1:** Detection frequencies of *S. mutans* in children with S-ECC were significantly higher than those in caries-free (CF) children (*p* < 0.05).

Groups	Number	Strains of *S. mutans*			
		Number (%)	Detection Frequency (%)	dmfs	*p*
S-ECC	35	63 (53.4)	92.4	9.3 ± 5.3	0.005
CF	32	55 (46.6)	62.5	0	
Total	67	118			

S-ECC, severe early-childhood caries; CF, caries-free; dmfs, decayed, missing, and filled surfaces of primary teeth.

### 3.2. Effect of Sucrose Concentration on the Synthesis of WIG

**Figure 1:**
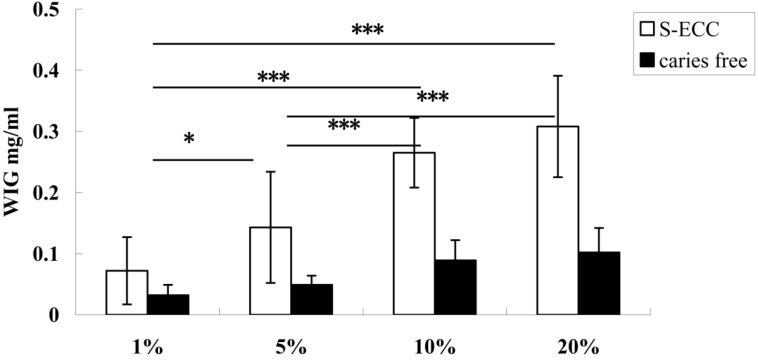
WIG synthesis of *S. mutans* from children with ECC and caries-free children with 1%, 5%, 10%, and 20% sucrose. White bars and black bars represent the means (with standard deviations) of WIG synthesized by *S. mutans* isolated from children with ECC and caries-free children. * Denotes statistical significance (*p* < 0.05), and *** Denotes statistical significance (*p* < 0.001).

According to the results shown in Figure 1, the synthesis of WIG increased significantly with the sucrose concentration, increasing from 1% to 10%. When the sucrose concentration was above 10%, the synthesis of WIG increased slowly. The highest WIG synthesis, found in the 20% sucrose group, did not differ significantly from that in the 10% sucrose group but was significantly higher than that in the 1% and 5% sucrose groups (*p* < 0.05).

### 3.3. Effect of Sucrose Concentration on the Adhesive Ability of S. mutans

**Figure 2:**
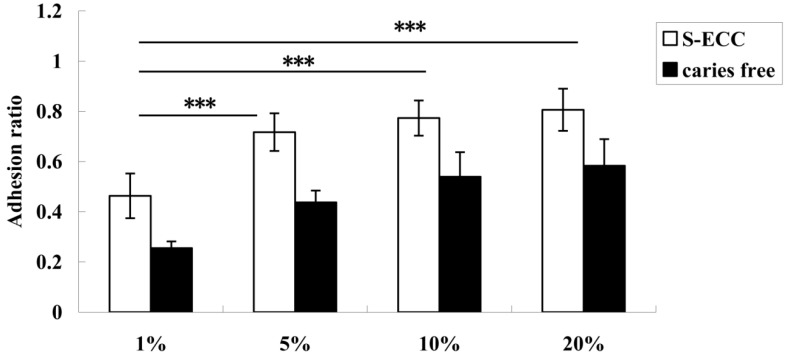
Adhesion ratios of *S. mutans* from children with ECC and caries-free children when the sucrose concentration was 1%, 5%, 10%, and 20%. The adhesion ratio in the 1% group was significantly lower than that in the 5%, 10%, and 20% groups. There were no significant differences among the 5%, 10%, and 20% groups. *** Denotes statistical significance (*p* < 0.001).

The adhesion ratio of *S. mutans* isolated from both the ECC and caries-free groups was evidently enhanced when the sucrose concentration was from 1% to 5%. The adhesion ratio at 1% was significantly lower than that in the 5%, 10%, and 20% sucrose groups (*p* < 0.001). From 5% sucrose and above, no significant differences were observed at all sucrose concentrations (Figure 2).

### 3.4. Effect of Sucrose Concentration on gtf Expression

**Figure 3:**
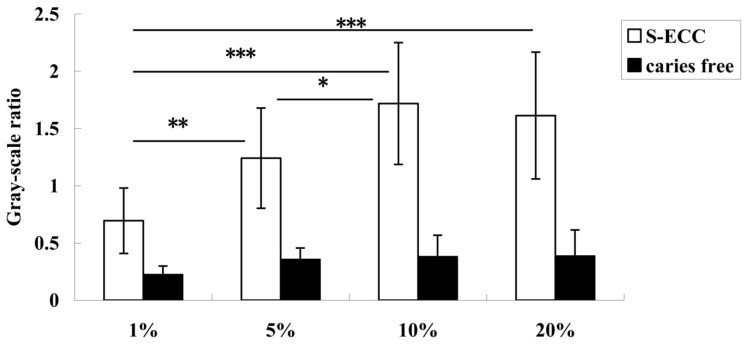
The expression of gtfC in *S. mutans* from children with ECC and caries-free children when the sucrose concentration was 1%, 5%, 10%, and 20%. The expression level of gtfC increased significantly when the sucrose concentration was 1% to 10%, and the 1% group expression was significantly lower than in the 5%, 10%, and 20% groups. * Denotes statistical significance (*p* < 0.05), ** Denotes statistical significance (*p* < 0.01), and *** Denotes statistical significance (*p* < 0.001).

**Figure 4:**
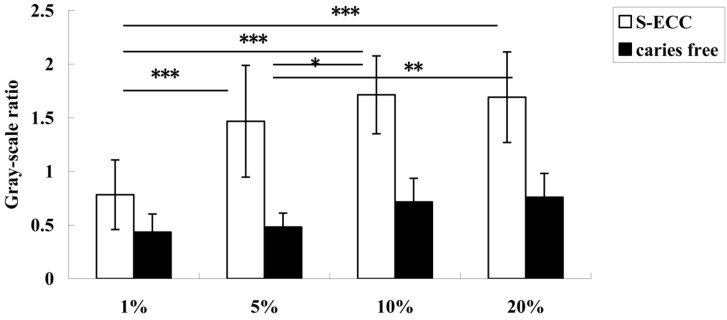
The expression of gtfB in *S. mutans* from children with ECC and caries-free children when sucrose concentration was 1%, 5%, 10%, and 20%. The expression level of gtfB increased significantly when the sucrose concentration was 1% to 10%, and the expression in the 1% and 5% groups was significantly lower than in the 10% and 20% groups. * Denotes statistical significance (*p* < 0.05), ** Denotes statistical significance (*p* < 0.01), and *** Denotes statistical significance (*p* < 0.001).

The relative mRNA expression levels of both gtfC (Figure 3) and gtfB (Figure 4) were sucrose-concentration-dependent. The highest gtfB and gtfC expressions were found in the 10% sucrose group. The expression level decreased when the sucrose concentration was above 10%, but there was no significant difference between the 10% and 20% sucrose groups. Therefore, 10% sucrose can be considered the “turning point” (Figure 3).

### 3.5. The Capacity for Sucrose-Dependent Adhesion of S. mutans from S-ECC and Caries-Free Groups

**Figure 5:**
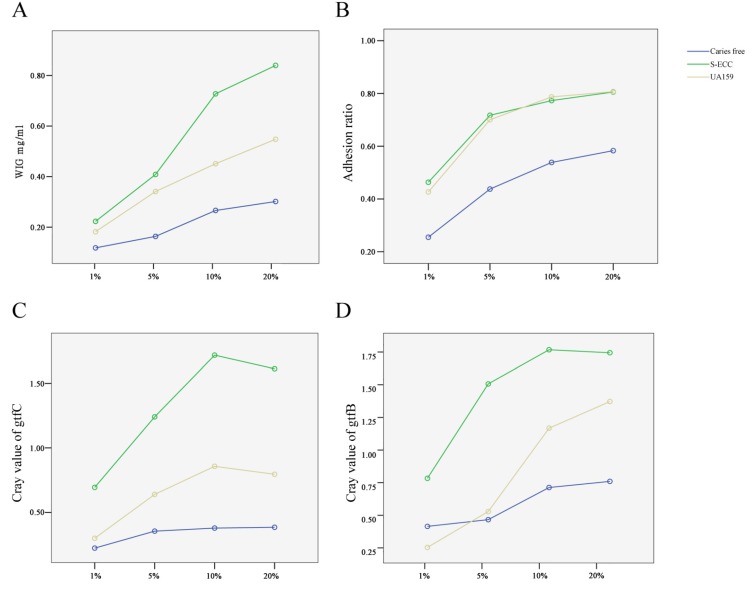
The capacity for sucrose-dependent adhesion of *S. mutans* from S-ECC and caries-free groups, with strain UA159 as control. The increase in WIG synthesis (**A**); adhesionratio (**B**); and the expression of gtfC (**C**) and gtfB (**D**) was sucrose-concentration-dependent, especially the strains from the S-ECC and UA159 groups.

To investigate whether different strains of *S. mutans* and different sucrose concentrations interacted during sucrose-dependent adhesion, we compared the capacity for sucrose-dependent adhesion of *S. mutans* from both S-ECC and CF groups, with strain UA159 used as positive control. As shown in Figure 5, the WIG synthesis, adhesion ratio, and the gene expression levels of gtfB and gtfC were enhanced with increased sucrose concentration and were dose-dependent. This tendency was more obvious in the S-ECC and the UA159 control groups. The capacity for sucrose-dependent adhesion of the strain UA159 detected in this study was higher than that of the strains from the CF group.

Using the general linear model and multifactor variance analysis, we compared the capacity for sucrose-dependent adhesion of *S. mutans* from the S-ECC and caries-free groups. The results indicated that the synthesis of WIG, adhesion ratio, and gene expression of gtfB and gtfC in *S. mutans* isolated from the CF group were significantly lower than those from the S-ECC (*p* < 0.01) group. The results confirmed that the virulence of *S. mutans* from children with S-ECC and caries-free children differed with regard to the synthesis of WIG and gtf expression.

### 3.6. Genotypes of S. mutans from the ECC and Caries-Free Groups

**Figure 6:**
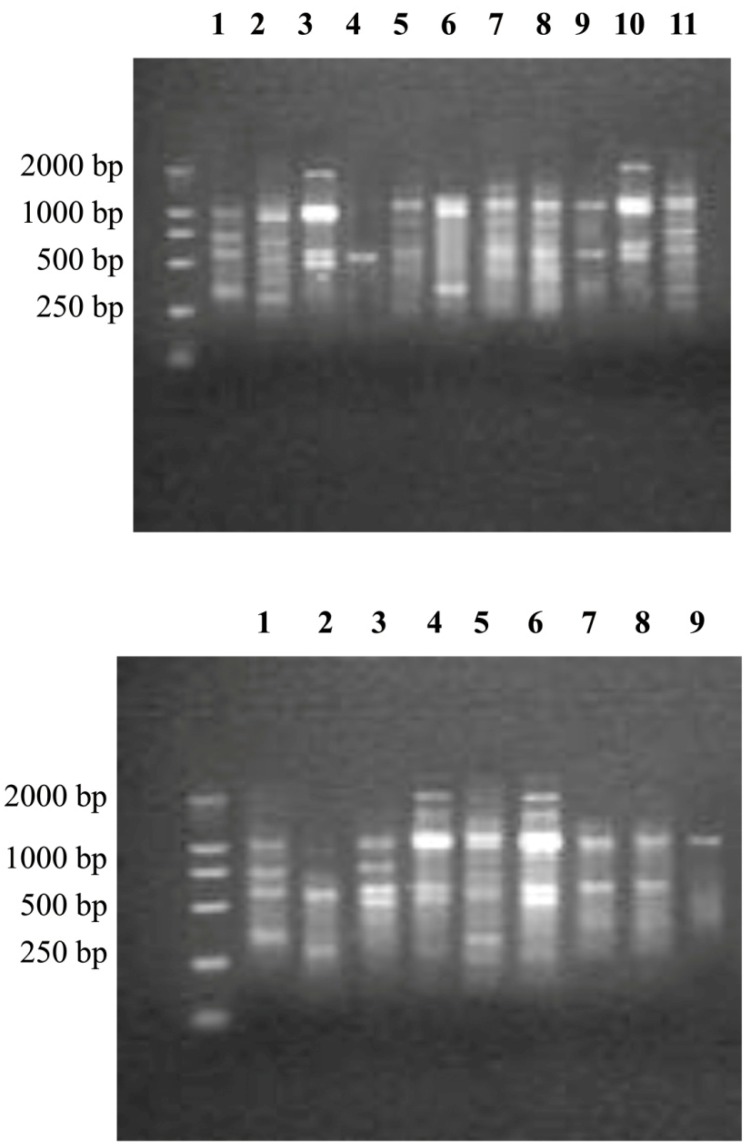
The genotypes of *S. mutans* from children with S-ECC (**A**) and caries-free (**B**) children. From 1 to 5 genotypes were detected in the ECC and caries-free groups, but the genotypes differed between the two groups.

From 1 to 5 genotypes of *S. mutans* were detected in both the S-ECC and CF groups. As shown in Table 2, 87.3% of children with S-ECC carrying *S. mutans* harbored more than one genotype, whereas 71% of the CF children carrying *S. mutans* harbored more than one genotype. Two genotypes of *S. mutans* accounted for the greatest proportion in the S-ECC and CF groups. However, according to the Mann–Whitney U test (Table 2), the fingerprints of *S. mutans* detected from individuals in the S-ECC group exhibited greater diversity than those from CF individuals (*p* < 0.01). Furthermore, it is interesting that most strains of *S. mutans* in both the S-ECC and CF groups displayed 500-bp and 1000-bp bands, although the genotypes were completely different (Figure 6).

**Table 2:** The genotypes of *S. mutans* from children with S-ECC and CF children. From 1 to 5 genotypes were detected in both groups.

Groups	Number (%)					
	1 Genotype	2 Genotypes	3 Genotypes	4 Genotypes	5 Genotypes	*p*
S-ECC	8 (12.7)	20 (31.7)	17 (27)	13 (20.6)	5 (7.9)	0.003
Caries-free	16 (29)	19 (34.5)	12 (21.8)	6 (10.9)	2 (3.6)	

### 3.7. The Relationship of Genotypes and gtf Gene Expression

To determine whether the gene expression of gtfB and gtfC was related to diverse genotypes of *S. mutans*, we used multifactor variance analysis, and the results revealed that in the S-ECC group, the expression of gtfB and gtfC in *S. mutans* with 1 to 5 genotypes was significantly different (*p* < 0.05). However, in the CF group, the expression of gtfC was significantly different only among the 1- to 5-genotype groups (*F* = 51.971, *p* < 0.05). There were no significant differences in gtfB expression among the diverse-genotype groups (*F* = 0.342, *p* = 0.847).

## 4. Discussion

*S. mutans* is one of the most important pathogenic factors of ECC [23]. The cariogenicity of *S. mutans* is associated with their ability to colonize and accumulate on tooth surfaces, and the formation of sucrose-dependent adhesion plays a key role in the process. Water-insoluble glucans (WIG) synthesized by gtf constitute the major part of the matrix of sucrose-dependent biofilm and provide binding sites for *S. mutans* for colonization and adherence. Therefore, WIG and gtf are considered the virulence factors of *S. mutans*. Previous studies have demonstrated that the differences in the capacities for WIG synthesis by *S. mutans* correlate with the caries experience of the children [8]. Thus, *S. mutans* strains appear to differ with regard to their gtf-mediated virulence. In the current study, we compared WIG synthesis, adhesion ability, and gtf gene expression of 63 strains of *S. mutans* from children with S-ECC with those of the other 55 strains from CF children. The results confirmed that the sucrose-dependent adhesion of *S. mutans* from children with ECC was significantly higher than that from caries-free children at the same sucrose level. Furthermore, they also proved that the virulence of *S. mutans* isolated from children with ECC is determined by WIG synthesis and gtf expression.

*S. mutans* produces at least three genetically separate GTFs, each of which synthesizes a structurally distinct glucan from sucrose. gtfB synthesizes primarily α-1,3-insoluble glucan. gtfC produces a mixture of α-1,6-soluble glucans and insoluble glucans, and gtfD forms predominantly soluble glucans [24,25,26]. The genes coding for each gtf isozyme were inactivated, and the resulting mutant strains were analyzed for sucrose-dependent adherence *in vitro* and for cariogenicity in animal models. These studies indicated that gtfB and gtfC have the highest association with virulence [27,28]. Factors such as carbohydrate (glucose or sucrose) exposure and environmental pH, which control the expression of gtfB and gtfC, may account for differences in virulence among infecting *S. mutans* strains. The results from some studies have demonstrated that sucrose induces the expression of gtfB [12,14,29]. However, other investigators have reported decreased expression of gtfB and gtfC [30].

Sucrose has been proven to be one of the most important virulence factors in the progression of S-ECC. The role of sucrose in dental caries has been investigated in biofilm models [19,31,32], *in vitro* models, *in situ* models, and animal models. The *in situ* model is composed of healthy adult volunteers who wear acrylic intraoral palatal appliances containing dental enamel blocks and add 1%, 5%, 10%, 20%, or 40% sucrose solution, 8 times a day [4,5,6,7]. This method can mimic the environment of the oral cavity. However, it cannot be applied in children’s mouths. The research done at the biofilm level can mimic the development and progression of S-ECC. However, many investigators choose the standard strains of *S. mutans*, such as UA159, for *in vitro* biofilm formation. There are still some difficulties involved in the study of dental biofilm samples collected from children with S-ECC and CF children. Therefore, in this study, we chose the *in vitro* model.

In this study, the expressions of gtfB and gtfC were up-regulated with increased sucrose concentration. The expression levels of the 10% and 20% sucrose groups were significantly higher than those in the 1% and 5% groups (*p* < 0.01). Moreover, no difference was found between the 10% and 20% sucrose groups, consistent with the results of the effect of sucrose concentration on WIG synthesis. The 10% sucrose concentration is regarded as the “turning point” because the expressions of gtfB and gtfC declined when the sucrose concentration was above 10%. Shemesh *et al.*, found that the expression of gtfB in TY culture medium containing 40% sucrose was slightly lower than that in the medium containing 10% sucrose, whereas the expression of gtfC under 40% sucrose is higher than 10%. However, they found no significant difference between the two groups [15]. Therefore, the expressions of gtfB and gtfC cannot be enhanced or down-regulated when the sucrose concentration is higher than 10%.

According to the present study, sucrose concentration is a regulator of gtf expression, WIG synthesis, and adhesion ability of *S. mutans*. A series of *in situ* studies also found that the threshold of sucrose concentration necessary to form a cariogenic biofilm is 5%, while the cariogenic potential of 10% and 20% sucrose was the same [4]. In this study, WIG synthesis and gene expression of gtfB and gtfC in the 5% sucrose group was significantly lower than that in the 10% and 20% groups, and there were no significant differences between the 10% and 20% groups. One systemic review presented to inform WHO guidelines for caries prevention showed that caries was lower when free-sugar intake was less than 10% [33]. Although the result was slightly different from those in the *in situ* studies, it can be concluded that the sucrose concentration exceeding 5% has more cariogenic potential for both the adhesion of *S. mutans* and the formation of cariogenic biofilm. Therefore, reducing the intake of food containing sucrose concentrations above 5% could effectively prevent ECC.

The expressions of gtfB and gtfC differed greatly between the S-ECC and CF groups. One study found that substitution of sucrose induced down-regulated expression of gtfB, gtf, gbpB, and vicR of the UA159 strain in a biofilm model [32]. However, the factors regulating the expression of gtf are still unclear. A few studies have targeted the virulence traits of different genotypes of *S. mutans*. Alaluusua *et al.* [34] found that the polysaccharide synthesis ability of *S. mutans* isolates with different genotypes from caries-active children was different from that of CF children, although the difference was not significant. Another study found that there were more *S. mutans* genotypes with the increased ability to synthesize water-insoluble glucans in caries-active individuals [8,35]. DNA fingerprint analysis revealed that the genotypes of *S. mutans* from 3- to 4-year-old children displayed great genetic diversity. From 1 to 5 genotypes of *S. mutans* were detected as colonizing the oral cavities of children with S-ECC and CF children [36]. In this study, the results of arbitrarily primed PCR also found from 1 to 5 genotypes of *S. mutans* from the S-ECC and CF groups. The fingerprints of *S. mutans* detected from the individuals in the S-ECC group exhibited greater diversity than those from the CF individuals. More importantly, the expression of gtfB and gtfC in the S-ECC group was significantly different among the 1- to 5-genotype groups. This has not yet been reported. The diversity of genotypes was related to the gtf expression levels of *S. mutans*, which suggested that the different gtf coding sequences might result in different virulences of *S. mutans* strains*.* Therefore, sequence analysis of the gtf gene, especially the promoter and the operon, could help explain the regulation mechanisms of gtf expression and even find molecular markers of ECC, which could be used to screen for highly cariogenic strains of *S. mutans*.

## 5. Conclusions

S-ECC is a serious public health problem, especially in developing countries. Sucrose has been proven to be an important virulence factor in S-ECC. However, the mechanisms regulating the sucrose-dependent adhesion of *S. mutans* are still not clear. In this study, we found that the synthesis of water-insoluble glucan (WIG), capacity for adhesion, and gene gtf expression in *S. mutans* isolated from S-ECC were sucrose-concentration-dependent and increased dramatically when sucrose concentration was 1%–10%. Apart from this, we found that the fingerprints of *S. mutans* in the S-ECC group showed significant differences in diversity compared with those in the CF group. Therefore, it can be concluded that sucrose-dependent adhesion might be related to the diversity of genotypes of *S. mutans,* and that a 10% sucrose level can be considered a “turning point”. The results of this study will be of great help in S-ECC prevention and oral health promotion for children.
